# Artificial Intelligence Applications in Sickle Cell Retinopathy Imaging: Current Progress, Challenges, and Future Directions

**DOI:** 10.1155/joph/5579203

**Published:** 2026-02-20

**Authors:** Parim Shah, Hamza Ahmed Farah, Daniel J. Wisotsky, Paarth Nawani, Katherine Kovrizhkin, Eric R. Muir, Umar Mian, Tim Q. Duong

**Affiliations:** ^1^ Department of Radiology, Albert Einstein College of Medicine and Montefiore Health System, Bronx, New York, USA; ^2^ Departments of Radiology and Ophthalmology, University of North Carolina at Chapel Hill, Chapel Hill, North Carolina, USA, unc.edu; ^3^ Department of Ophthalmology and Visual Sciences, Albert Einstein College of Medicine and Montefiore Health System, Bronx, New York, USA

**Keywords:** AI, convolutional neural networks (CNNs), deep learning, fluorescein angiography, fundus photography, optical coherence tomography, retinal imaging, sickle cell disease

## Abstract

**Introduction:**

Sickle cell retinopathy (SCR) is a leading cause of vision loss in patients with sickle cell disease, but its detection and monitoring remain difficult due to heterogeneous retinal microvascular changes and reliance on expert interpretation. Artificial intelligence has shown success in retinal disease detection, classification, and staging on retinal images, achieving expert‐level performance. This review summarizes recent artificial intelligence progress in SCR imaging and highlights future opportunities for clinical translation.

**Materials and Methods:**

This review was conducted in accordance with the Preferred Reporting Items for Systematic Reviews and Meta‐Analyses (PRISMA) guidelines. A comprehensive literature search was performed in PubMed/MEDLINE, Embase, and Web of Science.

**Results:**

A comprehensive PubMed search returned 15 records. After removal of duplicates and screening of titles and abstracts, 7 full‐text articles were assessed for eligibility. Of these, 4 studies met inclusion criteria and were included in the final review. Three full text articles were assessed for eligibility and inclusion from non‐PubMed sources. Two studies applied classical machine learning on features extracted from imaging data to classify SCR. Four studies used deep‐learning algorithms to detect SCR features on ophthalmological images. One study applied deep learning algorithms to classify SCR from other retinal diseases.

**Conclusions:**

Deep learning has the potential to improve detection, stage, and monitor sickle cell retinopathy across multiple ophthalmological imaging methods. Additional research is needed to support clinical adoption. With continued development, AI‐based tools could enhance diagnostic precision, enable personalized care, and ultimately improve outcomes for patients with sickle cell retinopathy.

## 1. Introduction

**Figure 1:**
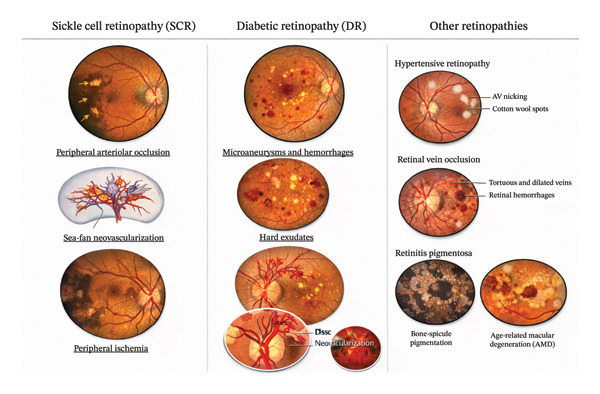
Representative imaging features of sickle cell retinopathy (SCR) compared with diabetic retinopathy (DR) and other common retinopathies. Fundus photographs and schematic illustrations demonstrate hallmark manifestations of SCR, including peripheral arteriolar occlusion, sea‐fan neovascularization, and peripheral ischemia, contrasted with typical features of DR such as microaneurysms, intraretinal hemorrhages, hard exudates, and neovascularization at the disc or elsewhere. Selected examples of other retinopathies illustrate overlapping but distinct vascular and structural abnormalities. The figure emphasizes disease‐specific patterns relevant to both clinical interpretation and AI‐based image analysis.

Sickle cell retinopathy (SCR) is a common and vision‐threatening​ complication of sickle cell disease, resulting from chronic vaso‐occlusion, ischemia, and progressive microvascular remodeling of the retina [1, 2]. Unlike more prevalent retinal vascular diseases such as diabetic retinopathy (DR), SCR is characterized by a predilection for peripheral ischemia, arteriolar occlusion, and the development of pathognomonic sea‐fan neovascularization in advanced stages (Figure 1). These features often precede macular involvement and may remain clinically silent until late in the disease course, increasing the risk of delayed diagnosis and irreversible vision loss. Accurate detection, staging, and longitudinal monitoring of SCR are therefore critical for guiding surveillance intervals and timely intervention.

Ophthalmic imaging is central to the evaluation and management of SCR, yet its interpretation remains challenging and variable. Ultra‐widefield (UWF) fundus photography and fluorescein angiography (FA) are essential for visualizing peripheral nonperfusion and neovascularization, while optical coherence tomography (OCT) and OCT angiography (OCTA) enable quantitative assessment of macular thinning, foveal avascular zone (FAZ) abnormalities, and microvascular density. However, SCR imaging findings are often subtle, spatially heterogeneous, and highly dependent on disease stage, making visual interpretation difficult and susceptible to substantial inter‐ and intraobserver variability [3, 4]. Manual grading is time‐consuming, largely qualitative, and heavily reliant on specialized retinal expertise that may be limited in resource‐constrained settings, resulting in poor reproducibility across readers and institutions [5, 6]. These challenges are further compounded by the fact that SCR manifestations differ fundamentally from those of DR and other retinal vascular diseases. Misclassification can therefore have important clinical consequences, as surveillance strategies, treatment thresholds, and prognostic expectations differ substantially between conditions; for example, proliferative SCR may require intensified peripheral monitoring despite relatively preserved central vision, whereas DR management is often driven by posterior pole findings. Failure to recognize SCR‐specific imaging patterns may delay referral, underestimate disease severity or lead to suboptimal follow‐up intervals. Collectively, these limitations create critical diagnostic gaps in standardization, scalability, and objective quantification, undermining accurate risk stratification and longitudinal assessment.

Artificial intelligence (AI)–based approaches [7] offer a compelling strategy for addressing the interpretive and scalability challenges inherent to SCR imaging by enabling automated, reproducible, and quantitative analysis across imaging modalities, readers, and clinical settings [7–9]. In ophthalmology more broadly, AI has demonstrated expert‐level performance in the detection, classification, and staging of retinal diseases such as DR, age‐related macular degeneration, and retinopathy of prematurity, establishing a strong precedent for its application to SCR. AI is especially well suited to the distinctive imaging characteristics of SCR, which include peripheral‐dominant pathology, subtle and spatially heterogeneous macular changes, and volumetric patterns across adjacent OCT B‐scans that are difficult to integrate consistently through manual review. By quantifying vascular features, detecting faint or distributed lesions, and leveraging volumetric and multimodal data at scale, AI models have the potential to reduce observer variability, improve diagnostic accuracy, and enable earlier detection and prognostic assessment. Moreover, automated approaches may support screening and triage in settings with limited access to retinal specialists, thereby addressing critical gaps in standardization and equity of eye care for populations disproportionately affected by sickle cell disease.

AI as an umbrella term referring to computational systems designed to perform tasks that typically require human intelligence, such as pattern recognition, classification, and decision support. Machine learning (ML) is a subset of AI in which algorithms learn statistical relationships directly from data rather than relying on explicitly programmed rules. Deep learning (DL) is a further subset of ML that employs multilayer neural networks capable of learning hierarchical feature representations from high‐dimensional inputs, including medical images. Explainable AI refers to a complementary set of methods applied to AI, ML, and DL models to improve interpretability and transparency by providing insight into model reasoning, feature importance, or decision pathways (such as saliency maps), particularly in clinical settings where trust and accountability are essential. Classical ML algorithms, including support vector machines (SVM), random forests, and gradient boosting, rely on engineered features such as vessel tortuosity, FAZ metrics, and parafoveal density to learn discriminative patterns [10, 11]. While interpretable and suitable for small datasets, these methods are often limited by the quality and completeness of handcrafted features [12]. DL approaches, particularly convolutional neural networks (CNNs), hierarchical residual networks (ResNets), and vision transformers (ViTs), can employ end‐to‐end representation learning, automatically capturing multiscale spatial and textural features from raw imaging data [13–15]. Transformer‐based architectures and attention mechanisms further enable modeling of long‐range dependencies, enhancing localization and interpretability of subtle pathological changes [13, 16, 17]. Hybrid frameworks augment these capabilities through multimodal integration and domain‐specific priors such as artery–vein differentiation or vascular feature fusion, while strategies including contrastive learning and self‐supervised pretraining learning improve performance on limited and imbalanced datasets [18, 19]. Collectively, these advanced AI methods enable highly scalable, precise, and clinically relevant characterization of complex retinal pathologies such as SCR.

In this review, we systematically summarize and critically evaluate recent applications of AI to SCR imaging. We examine how different AI methodologies have been applied across imaging modalities, assess their reported performance and limitations, and synthesize common challenges that currently hinder clinical translation. Finally, we highlight key opportunities for future research, including multimodal integration, longitudinal prediction, robust external validation, and the development of interpretable models aligned with clinically meaningful endpoints.

## 2. Materials and Methods

### 2.1. Search Strategy

No ethics committee approval was required for this literature review. This review was conducted in accordance with the Preferred Reporting Items for Systematic Reviews and Meta‐Analyses (PRISMA) guidelines. A comprehensive literature search was performed in PubMed/MEDLINE, Embase, and Web of Science, to identify relevant studies published from inception through December 1, 2025. The search strategy combined controlled vocabulary and free‐text terms related to SCR and AI, including variations of search terms’ combined concepts for SCR (including “sickle cell retinopathy,” “sickle cell disease AND retina,” “sickle cell maculopathy,” and the medical subject headings (MeSH) terms Anemia, Sickle Cell with Retina or Macula Lutea) with terms for AI methods (including “artificial intelligence,” “machine learning,” “deep learning,” and “computer‐aided”). This strategy was designed to capture studies that applied computational or automated approaches specifically to SCR. Reference lists of included articles and relevant reviews were also manually screened to identify additional eligible studies.

### 2.2. Eligibility Criteria

Reference lists of included papers were also screened to capture additional relevant articles not identified by database queries. Studies were included if they (1) involved patients with sickle cell disease or SCR; (2) reported on AI, ML, or quantitative image analysis; and (3) used fundus photography, FA, or OCT. Exclusion criteria were (1) studies not involving human subjects, (2) articles not in English, and (3) studies focused on ophthalmic diseases other than SCR. The initial search and screening were performed independently by two reviewers (PS and PN), with discrepancies resolved by discussion and consensus.

### 2.3. Study Selection

All identified records were imported into a reference management system, and duplicates were removed. Two reviewers independently screened titles and abstracts for relevance, followed by full‐text review of potentially eligible studies. Discrepancies were resolved by consensus or by consultation with a third reviewer. The study selection process is summarized using a PRISMA flow diagram.

### 2.4. Data Extraction

Data were independently extracted by two reviewers using a standardized data extraction form. Extracted variables included study design, population characteristics, imaging modality, AI task (e.g., detection, grading, and prediction), model type (e.g., classical ML and CNN), input features, reference standard, dataset size, validation strategy, performance metrics, and use of explainability techniques. Any disagreements in data extraction were resolved through discussion.

### 2.5. Data Synthesis and Comparison of AI Studies

Given the heterogeneity of study designs, imaging modalities, AI architectures, and outcome measures, a quantitative meta‐analysis was not performed. Instead, results were synthesized qualitatively. Studies were grouped and compared according to clinical task, imaging modality, and AI methodology. We specifically evaluated how each study addressed known clinical limitations of manual SCR interpretation, such as observer variability, scalability, and sensitivity to early disease. Performance metrics and validation approaches were summarized to facilitate comparison, and the role of explainable AI techniques was noted where applicable.

## 3. Results

**Figure 2:**
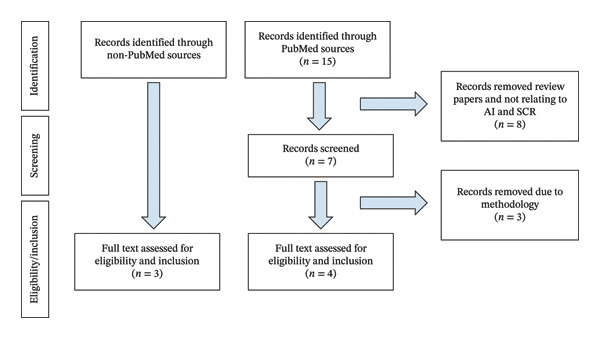
PRISMA selection flowchart.

Figure 2 shows the PRISMA selection flowchart. A comprehensive PubMed search returned 15 records. After removal of duplicates and screening of titles and abstracts, 7 full‐text articles were assessed for eligibility. Of these, 4 studies met inclusion criteria and were included in the final review. Three full text articles were assessed for eligibility and inclusion from non‐PubMed sources.

Table 1 summarizes the AI applications to SCR on ophthalmological images. Two studies applied classical ML on features extracted from imaging data to classify SCR. Alam et al. [20] analyzed 70 SCR eyes (29 mild and 6 severe) and 14 controls using six features: blood vessel tortuosity (BVT), vessel diameter, vessel perimeter index, FAZ area, FAZ contour irregularity, and parafoveal avascular density (PAD). SVM achieved 100% accuracy for SCR vs control and 97% for mild vs severe, outperforming k‐nearest neighbor (95%) and discriminant analysis (88%). BVT, FAZ area, and PAD were the most sensitive features, demonstrating the utility of ML for SCR classification. This work demonstrated a ML approach to classify SCR vs normal retinal images (binary classification) with strong internal performance across classifiers. However, this is a small and single‐center, used both eyes, and had no patient‐level split or outside test set.

**Table 1:** AI and sickle cell retinopathy.

Study	Modality/device	Imaging features	Sample size (pts/eyes/images)	Population characteristics	Objective	AI techniques	Performance
Alam et al. [20]	OCTA/AngioVue SD‐OCT (Optovue)	BVT, BVC, VPI, BVD, FAZ area, FAZ contour irregularity	48 pts/90 eyes/90 images (30 mild SCR, 18 severe SCR)	SCR patients; 90% African American; HbSS and HbSC	Stage SCR (mild vs severe)	SVM	Sensitivity: 93.19%, specificity: 91.60%, AUC: 0.97
Alam et al. [10]	OCTA/AngioVue SD‐OCT (Optovue)	BVT, BVC, VPI, BVD, FAZ area, FAZ contour irregularity	60 DR pts, 48 SCR pts, 20 controls/115 DR images, 90 SCR images, 40 control images	DR: mild (20), moderate (20), severe NPDR (20); SCR: mild (30), severe (18); controls (20)	Classify (1) normal vs disease, (2) DR vs SCR, (3) stage DR, (4) stage SCR	Hierarchical multitask SVM	Control vs disease: AUC 0.98; DR vs SCR: AUC 0.94; NPDR staging: AUC 0.96; SCR staging: AUC 0.97
Cai et al. [21]	UWF‐CFP/Optos 200Tx	Sea‐fan neovascularization	190 pts/336 eyes/1182 images	HbSS (62.6%), HbSC (24.2%);94.2% Black/African descent	Detect sea‐fan neovascularization from UWF‐CFP	CNN (Inception V4)	Sensitivity: 97.4%, specificity: 97.0%, AUC 0.988
Jin et al. [22]	OCT B‐scans/N/A	Inner‐retinal thinning	33 pts/63 OCT studies/3906 B‐scans	Children with SCD; 14 males; 21 HbSS, 9 HbSC	Detect retinal thinning from SCR and differentiate from normal fovea	YOLO grid‐based object detection	Fovea detection: mAP 96%; SCR detection: mAP 72%
Bhattarai et al. [23]	OCT B‐scans/N/A	Inner‐retinal thinning across multiple B‐scans	90 pts/331 OCT studies/9,320 sets of 3 adjacent B‐scans (for detector training); 47,787 positive and 337,700 negative pairs (for pre‐training)	Children with SCD	Differentiate subtle SCR changes from artifacts using adjacent scans as reference	Detachable encoder transformer (DEnT): (1) pre‐trainer using Siamese network, (2) detector using transformer	SCR detection mAP: 0.86
Abitbol et al. [24]	OCT B‐scans/N/A	Irregularities in cross‐sectional images	Pre‐trainer: 385,487 scan pairs; detector: 9,320 triplets from 331 OCT (90 pts)	Children with SCD	Identify SCR irregularities in cross‐sectional OCT	Cross scan attention transformer (CSAT): Siamese pre‐trainer, transformer‐based detector	SCR detection mAP: 0.86
Cai et al. [25]	OCT B‐scans/N/A	Overall image patterns for disease classification	224 total images: 57 SCR, 65 DR, 47 RVO, 55 healthy controls	N/A	Classify SCR, DR, RVO, and healthy controls	CNN (DenseNet121)	SCR: accuracy 93.8%, AUC‐ROC 96.7%, Sens 94.7%, Spec 93.4%

*Note:* UWF‐CFP = ultra‐widefield color fundus photography; DEnT = detachable encoder transformer; HbSC = hemoglobin SC disease; HbSS = hemoglobin SS disease; pts = patients.

Abbreviations: BVC = blood vascular caliber, BVD = blood vessel density, BVT = blood vessel tortuosity, CNN = convolutional neural network, CSAT = cross scan attention transformer, DR = diabetic retinopathy, FAZ = foveal avascular zone, mAP = mean average precision, N/A = not available, NPDR = nonproliferative diabetic retinopathy, OCT = optical coherence tomography, OCTA = optical coherence tomography angiography, RVO = retinal vein occlusion, SVM = support vector machine, VPI = vessel perimeter index, YOLO = You Only Look Once.

Alam et al. [20] developed a vessel‐aware, quantitative OCTA analysis framework for SCR classification that incorporated a hierarchical multitask SVM classifier. Their dataset included 205 eyes (90 SCR, 20 controls, and remainder DR), and the model was trained to distinguish between three key tasks: normal vs. disease, SCR vs. DR, and stage I vs. stage II SCR. Using artery–vein classification and vessel‐specific metrics such as caliber, tortuosity, and artery–vein ratios, the approach achieved high discriminative performance. For disease vs. control, the AUCs were 0.98, 0.97, and 0.98 across different test configurations; for stage I vs. stage II SCR, performance was similarly strong with AUCs of 0.93, 0.92, and 0.97. These results demonstrate that artery/vein‐specific quantitative OCTA features, combined with hierarchical multitask SVM modeling, provide a robust framework for distinguishing SCR from controls, differentiating SCR from DR, and staging SCR.

Four studies used DL algorithms to detect SCR features on ophthalmological images. Cai et al. [21] trained an Inception V4 CNN, a DL approach, on 1,182 UWF fundus images from 190 patients (53% women, 94% Black/African descent, 63% hemoglobin SS genotype, and 24% hemoglobin SC genotype) to detect sea‐fan neovascularization, a hallmark of proliferative SCR (PSR). In this study, de‐identified UWF fundus photographs were independently graded by two masked retinal specialists for the presence or absence of sea‐fan neovascularization, with a third masked retinal specialist adjudicating discordant or indeterminate cases. These consensus grades served as the ground truth for model training. Data were split 70:20:10 into training, testing, and validation, respectively. The model, trained with binary cross‐entropy loss over 100 iterations, achieved area under the curve (AUC) 0.988, 97.0% accuracy, 97.4% sensitivity, and 97.0% specificity, matching or surpassing retinal specialists. Saliency maps (SmoothGrad and Guided Gradient‐weighted Class Activation Mapping) consistently localized pathology, addressing the “black box” limitation. Overall, UWF DL can spot sea fans at specialist level, but it still needs multisite/device testing, prospective testing, and broader PSR endpoints before screening use in clinical settings.

In a conference abstract, Jin 2022 used DL to analyze OCT B‐scans for detection of regions of inner‐retinal thinning caused by SCR. They used a You Only Look Once (YOLO) object detection algorithm. The dataset had a total of 3906 images from 63 OCT studies of 33 sickle cell disease pediatric patients with examples of SCR regions and fovea annotated by experts using bounding boxes. Dataset was divided into 5 equal sets for 5‐fold cross‐validation and used an 80:20 training and testing split with binary cross‐entropy and focal loss as metric for measuring training loss. Ultimately the model reached an average mean average precision (mAP) of 96% and 72% on fovea and SCR region detection, respectively. They attributed the lower mAP for the SCR detection to ambiguity and inconsistency in annotating SCR, something that has been stated by similar studies before. Therefore, YOLO can spot SCR‐related thinning on pediatric OCT, but the small single‐center data (63 studies and 33 patients), variable labels, inconsistency of SCR annotation, and lack of patient‐wise external testing mean results may not generalize.

Bhattarai et al. [22] used 594 OCT volumes from 147 sickle cell patients, and each image was annotated by experts for SCR features. To enhance detection beyond single scan approaches, they introduced a cross scan attention transformer (CSAT), designed to integrate adjacent B‐scans into a unified feature set. This architecture, pretrained with a ResNet50‐based Siamese network to emphasize retinal thickness and contour information, demonstrated superior performance compared with commonly used detectors. Performance was evaluated using mAP at different Intersection over Union (IoU) thresholds, where IoU measures the overlap between the predicted and expert‐annotated lesion boxes. Using cross‐scan attention, the CSAT achieved an mAP of 0.86 at a 50 percent threshold overlap and 0.83 at the stricter 50%–95% range, indicating both accurate and precise lesion detection. This performance surpassed that of commonly used detectors which scored lower at the same thresholds, including YOLOv8 (0.78, 0.72), faster region‐based CNN (R‐CNN) (0.78, 0.69), and detection transformer (DETR) (0.81, 0.76). While the CSAT results demonstrated improved detection of SCR from OCT images over single‐scan detectors, the lack of public data or external validation limits conclusions about its generalizability across devices and clinical situations.

Bhattarai et al. [23] in a conference abstract reported the use of the detachable encoder transformer (DEnT), a two‐phase DL approach to detect SCR on OCT images, leveraging the volumetric nature of OCT B‐scans. In the first phase, the DEnT pre‐trainer used a Siamese network with a contrastive learning framework to analyze pairs of augmented B‐scans, determining whether they originate from the same OCT study, thus learning to identify changes in retinal layer contour, shape, and thickness across scans. This pre‐trainer was trained on 47,787 positive and 337,700 negative B‐scan pairs. In the second phase, the DEnT detector used the pre‐trained encoder to process three adjacent B‐scans simultaneously and then applying positional encoding from one B‐scan to query features in the others. The detector was trained on 331 OCT studies from 90 sickle cell disease patients, comprising 9,320 sets of three adjacent B‐scans, with three models tested: one without pre‐training, one with pre‐training, and one with pre‐training and fine‐tuning. Although the abstract does not explicitly state the reference standard, performance was reported using mAP, which in comparable OCT detection studies reflects comparison against expert annotations of SCR lesions. The fine‐tuned model had mAP of 0.86 and 0.83 at IoU of 0.5 and 0.5–0.9 respectively, outperforming YOLOv8 (0.78, 0.72), Faster R‐CNN (0.78, 0.69), and DETR (0.81, 0.76) at the same thresholds. These results show that DEnT can detect SCR from volumetric B‐scans, but validation remains limited to single site internal data.

One study applied DL algorithms to classify SCR from other retinal diseases. Abitbol et al. [24] evaluated a DenseNet121 model trained on 224 UWF fundus images representing DR, retinal vein occlusion (RVO), SCR, and healthy eyes. Using cross‐validation, the model achieved an overall accuracy of 88.4%, with the strongest performance for SCR (AUC 0.967, accuracy 93.8%). In contrast, DR (AUC 0.905, accuracy 85.2%) and RVO (AUC 0.912, accuracy 88.4%) showed more overlap in misclassification. Visualization approaches, including saliency maps and Grad‐CAM++, highlighted clinically relevant regions, such as peripheral lesions in SCR and hemorrhagic patterns in DR and RVO. These results demonstrate that UWF imaging combined with DL can reliably differentiate SCR from other retinal vascular diseases, though larger, multisite validation will be necessary for clinical adoption.

## 4. Discussion

This review highlights the growing application of AI‐based methods to SCR, demonstrating that both classical ML and DL approaches can achieve strong performance across a range of imaging modalities and clinical tasks. Collectively, these studies suggest that AI has the potential to address key limitations of manual SCR interpretation, including variability in expert grading, limited scalability, and difficulty detecting subtle or peripheral disease features. In particular, the consistent success of vessel‐specific quantitative features, UWF imaging, and volumetric OCT‐based models underscores the importance of leveraging disease‐relevant retinal anatomy and spatial context rather than relying solely on single‐image classification.

From a clinical perspective, the reviewed work suggests several potential areas of impact. AI systems capable of reliably detecting proliferative features such as sea‐fan neovascularization could support screening and triage in populations with limited access to retinal specialists, while automated analysis of OCT and OCTA may enable earlier identification of ischemic or structural changes before overt proliferative disease develops. Furthermore, models designed to differentiate SCR from DR or other vascular retinopathies address a clinically relevant diagnostic challenge, particularly in patients with overlapping risk factors. However, these potential benefits remain largely theoretical at present, as none of the reviewed studies have yet demonstrated real‐world clinical integration or prospective impact on patient outcomes.

### 4.1. Limitations and Challenges

Despite promising proof‐of‐concept results, current AI applications in SCR imaging remain limited by methodological constraints that restrict generalizability and delay clinical translation. Although individual studies often acknowledge specific weaknesses, these limitations are rarely considered in aggregate, obscuring their combined impact on model robustness and reliability.

A primary concern is the dependence on small, single‐center datasets. Most models are developed using data from a single institution, imaging device, and acquisition protocol, which increases susceptibility to overfitting and limits confidence in performance beyond the development cohort. In the absence of demographic, geographic, and technical diversity, reported accuracy may reflect dataset‐specific characteristics rather than generalizable disease representations.

Annotation quality further complicates interpretation, particularly for OCT‐based detection of SCR‐related abnormalities. Subtle features such as inner‐retinal thinning and ischemia are spatially heterogeneous and challenging to delineate consistently, even among expert graders. While some studies acknowledge annotation uncertainty, few adopt standardized labeling protocols or consensus definitions of SCR imaging biomarkers. This lack of harmonization undermines reproducibility and complicates cross‐study comparisons.

Methodological choices in dataset construction and validation also introduce bias. Many analyses are performed at the image or eye level rather than the patient level, raising the risk of data leakage when multiple scans from the same individual are distributed across training and testing sets. Such practices can inflate performance metrics and do not reflect real‐world deployment, where models must generalize to entirely unseen patients. Moreover, validation is typically limited to internal cross‐validation or held‐out subsets, with little to no external, multi‐institutional testing.

Device and protocol dependence represents an additional barrier to translation. Models are often trained on images from a single OCT platform or UWF camera, making them sensitive to variations in resolution, field of view, and acquisition parameters. This limitation is particularly relevant in SCR, where peripheral pathology is central to disease staging and imaging practices vary widely across centers. Few studies explicitly evaluate robustness across devices or demonstrate consistency under heterogeneous imaging conditions.

Finally, most existing work focuses on cross‐sectional detection or classification. While automated identification of SCR features is an important first step, clinical care depends heavily on longitudinal assessment, including prediction of disease progression and risk of proliferative complications. The scarcity of longitudinal modeling and outcome‐based validation substantially limits the immediate clinical relevance of current AI approaches.

### 4.2. Future Directions

Overcoming these limitations will be essential for meaningful clinical translation of AI in SCR imaging. A critical priority is the creation of larger, multicenter datasets that capture the heterogeneity of SCR across populations, disease stages, and imaging protocols. Collaborative data‐sharing efforts will be particularly important for reducing bias and ensuring equitable model performance in a disease that disproportionately affects underserved populations [1, 25].

Integration of multimodal ophthalmic imaging represents another key opportunity. Clinical assessment of SCR routinely synthesizes information from OCT, OCTA, fundus photography, and UWF angiography, yet most AI studies analyze these modalities in isolation [26, 27]. Multimodal frameworks that fuse complementary biomarkers—such as macular thinning, ischemic indices, and peripheral lesion patterns—may substantially improve diagnostic accuracy and disease staging [28–30]. Future work should prioritize architectures that combine these inputs in clinically interpretable ways.

Emerging AI strategies also offer promising solutions to data and annotation challenges. Active learning can optimize expert labeling effort, while graph‐based models enable relational analysis of retinal vasculature. Few‐shot learning and generative adversarial networks (GANs) may help expand training datasets in rare or imbalanced settings [31–34]. Transfer learning and multimodal fusion approaches further allow models to leverage large external retinal datasets while incorporating SCR‐specific features such as FAZ morphology and OCT‐derived thickness measures [35–37].

Advances in DL architectures may further enhance robustness. Transformer‐based models, contrastive learning, and self‐supervised pretraining have demonstrated strong performance in ophthalmic imaging by leveraging unlabeled or weakly labeled data [38, 39]. These approaches are particularly well suited to volumetric OCT and OCTA, where spatial context across adjacent B‐scans carries important clinical information.

Beyond detection, future AI systems should emphasize longitudinal and predictive modeling. Clinical decision‐making in SCR depends on anticipating progression, proliferative risk, and the need for intervention. Models that analyze serial imaging over time, incorporate uncertainty estimation, and undergo prospective validation could support personalized surveillance and referral strategies [40, 41].

Finally, interpretability must remain central to AI development in SCR. Explainable AI techniques—including saliency mapping, attention visualization, and vascular feature attribution—are essential for clinician trust and workflow integration. Aligning model outputs with established and emerging SCR biomarkers, such as ischemic index, FAZ morphology, and quantitative vascular metrics, will help ensure that AI systems provide actionable insights rather than opaque predictions [42–44]. Ultimately, prospective, multisite studies evaluating clinical outcomes will be required to define the role of AI as a decision‐support tool in SCR care.

## 5. Conclusions

DL has the potential to improve detection, stage, and monitor SCR across multiple ophthalmological imaging methods. DL methods has the potential to match or surpass expert performance, offering scalable tools for screening and early detection. Additional research is needed to improve generalizability and interpretability and to support clinical adoption. With continued development, AI‐based tools could enhance diagnostic precision, enable personalized care, and ultimately improve outcomes for patients with SCR.

## Author Contributions

Parim Shah, Hamza Ahmed Farah, Umar Mian, and Tim Q. Duong participated in the design of the structure of the research manuscript. Parim Shah, Hamza Ahmed Farah, and Daniel J. Wisotsky contributed to the review of the systematic review and managed data collection. Parim Shah and Hamza Ahmed Farah drafted the manuscript. Paarth Nawani, Katherine Kovrizhkin, Umar Mian, Eric R. Muir, and Tim Q. Duong edited the manuscript. Umar Mian and Eric R. Muir contributed to clinical expertise. Tim Q. Duong supervised all authors who reviewed the final manuscript.

## Funding

No funding was received for this manuscript.

## Disclosure

The authors have nothing to report.

## Ethics Statement

The authors have nothing to report.

## Consent

The authors have nothing to report.

## Conflicts of Interest

The authors declare no conflicts of interest.

## Data Availability

This is a literature review paper. There are no data to share.
